# Improving Cognitive Visual-Motor Abilities in Individuals with Down Syndrome

**DOI:** 10.3390/s19183984

**Published:** 2019-09-14

**Authors:** Pablo V. Torres-Carrión, Carina S. González-González, Pedro A. Toledo-Delgado, Vanesa Muñoz-Cruz, Rosa Gil-Iranzo, Nuria Reyes-Alonso, Selene Hernández-Morales

**Affiliations:** 1 iHCI Research Group, Department of Computer Science, Universidad Técnica Particular de Loja 110160, Ecuador; pvtorres@utpl.edu.ec; 2 ITED Research Group, Department of Computer Science and Engineering, Universidad de La Laguna, 38200 San Cristóbal de La Laguna, Tenerife, Spain; petode@ull.edu.es (P.A.T.-D.); vmunoz@ull.edu.es (V.M.-C.); 3 Department of Computer and Industrial Engineering, Universidad de Lleida, 25001 Lleida, Spain; rosa.gil@gmail.com; 4 Asociación Tinerfeña de Trisómicos 21, Down Tenerife, 38204 La Laguna, Tenerife, Spain; nuria1701@hotmail.com (N.R.-A.); fisiodarias@gmail.com (S.H.-M.)

**Keywords:** gestural interaction, rehabilitation, down syndrome, KINECT sensor, exergames

## Abstract

Down syndrome causes a reduction in cognitive abilities, with visual-motor skills being particularly affected. In this work, we have focused on this skill in order to stimulate better learning. The proposal relies on stimulating the cognitive visual-motor skills of individuals with Down Syndrome (DS) using exercises with a gestural interaction platform based on the KINECT sensor named TANGO:H, the goal being to improve them. To validate the proposal, an experimental single-case study method was designed using two groups: a control group and an experimental one, with similar cognitive ages. Didactic exercises were provided to the experimental group using visual cognitive stimulation. These exercises were created on the TANGO:H Designer, a platform that was designed for gestural interaction using the KINECT sensor. As a result, TANGO:H allows for visual-motor cognitive stimulation through the movement of hands, arms, feet and head. The “Illinois Test of Psycholinguistic Abilities (ITPA)” was applied to both groups as a pre-test and post-test in its four reference sections: visual comprehension, visual-motor sequential memory, visual association, and visual integration. Two checks were made, one using the longitudinal comparison of the pre-test/post-test of the experimental group, and another that relied on comparing the difference of the means of the pre-test/post-test. We also used an observational methodology for the working sessions from the experimental group. Although the statistical results do not show significant differences between the two groups, the results of the observations exhibited an improvement in visual-motor cognitive skills.

## 1. Introduction

Down syndrome (DS) is a genetic disorder that causes a reduction in cognitive abilities to varying degrees. The cognitive visual-motor ability (CVMA) is one of the most sensitive, and it is a fact that this cognitive ability can be improved in people with DS [1,2,3,4]. In general, CVMA refers to the ability to integrate vision with the motor system, a requirement to comply with visually guided motor actions. This visual-motor capacity has been under-stimulated in the areas of formal education in children with DS [5], despite the continuous development of new technologies that facilitate gestural interactions with computer programs through hands, arms, legs, face, fingers, and body motility in general, as detailed in the ISO 9241-960 standard [6]. For example, there are spaces for gestural interaction using tablets that require the individual to select learning objects in interactive environments by means of natural gestures [7,8].

The relationship between working memory (WM), learning, and visual-motor memory has been extensively studied. Deficits in WM and visual-motor integration are explained based on neurological mechanisms, which are common in people with attention deficit/hyperactivity disorder (ADHD), autism [9], and Down Syndrome [10,11]. Working Memory and Monitoring as executive functions can predict visual-motor integration skills in children with intellectual disability [12] and with Down Syndrome, with relevant results due to sustained learning abilities in their visual-motor skills [1,2,3,13]. Of note among the studies carried out in the USA from 1990 to 2016 is the use of tests to assess CVMA [14], with the main focus being the population with special abilities: (a) people with higher degrees of disability, such as older adults [15], and people with motor and cognitive disabilities and (b) people with high physical abilities (elite athletes) and cognitive abilities. Due to its tools, the ImPACT clinical report (Verbal Memory, Visual Memory, Visual Motor Speed, and Reaction Time) is applied in most studies involving athletes [16,17,18]. For our study, in consideration of the objectives set, we decided to apply the Illinois Test of Psycholinguistic Abilities (ITPA) test, selecting the four sub-tests corresponding to CVMA: visual comprehension, visual association, visual integration, and visual-motor sequential memory. Stimulating the CVMA has yielded improved learning processes, with greater effort from the scientific community in people with disabilities and giftedness. In this same line of research requirements, the interactive environments seek to integrate tools to promote a better user experience, with non-invasive sensors and multiple forms of stimulation. A favorable response to stimulation has also been obtained with audio, regarding working memory and visual-motor areas [19] and gross motor skills [20] in an environment of natural interaction.

For the reasons explained above, the objective of this research is to evaluate if the gestural interaction with the KINECT sensor improves the cognitive visual-motor abilities in individuals with Down syndrome (DS). To this end, we designed thought and visual reasoning exercises as part of the activities of selective, discriminative and classification application, which allow training the cognitive abilities of attention, reasoning, and logic [21]; they are visible in the working memory model of Baddeley and Hitch [22], where both verbal and visual-spatial information require separate systems for their short-term maintenance, requiring didactic strategies parallel from these two systems to promote better learning. From this context and referring to the population with DS, stimulating the CVMA is strategic to enhancing learning. For the purposes of this study, and based on the stimuli applied during the experimentation phase in the gestural interaction platform, four sub-tests were selected: visual comprehension, visual association, visual integration, and visual-motor sequential memory.

Furthermore, therapists use exergames for rehabilitation to motivate patients to perform the exercises of their treatment [23,24,25,26]. The bibliography contains studies on the benefit of this motivation, regardless of the type of patient, illness, age, etc. [27]. Although there are several related papers that contain classifications of game characteristics for rehabilitation and applications using KINECT [28,29,30,31,32], we need to study the rehabilitation of cognitive visual-motor abilities using this sensor. This paper is organized as follows. In Section 2, we present some relevant related works on CVMA and DS, then materials and methods. The TANGO:H platform is described in Section 3. The validation is presented in Section 4. Finally, we offer a discussion in Section 5 and the conclusions in Section 6.

## 2. Related Works

Several studies have been carried out to stimulate and measure CVMA in a population with DS [33,34,35]. Cognitive and motor abilities have been evaluated using the Non-Verbal Intelligence Test (TONI) designed for subjects between six and 10 years old [36] as a measurement and validation tool in the methodological context; Chapman et al. [33] measured the performance at the beginning of the study and growth trajectory, adjusting the linear growth curves for simultaneous measurements of each individual of non-verbal visual cognition (Pattern Analysis subtest of the Stanford Binet), visual short-term memory (Bead Memory subtest), and auditory short-term memory/digit span [33].

Vicari et al. [37] used the episodic explicit memory test for visual-perception material (Spatial Sequences Learning with Corsi supraspan; explicit recognition of material studied in the Fragmented Pictures Test) together with other specific tests to expose the functional dissociation in people with DS, demonstrating that with the techniques that stimulate implicit memory results comparable to children with typical development of equal cognitive age; objects that require visual-perceptual processing were part of these stimulus tools [36]. Later, Vicardi et al. [38] corroborated these results with tasks that involve stimuli with spatial data processing, without significant difference in the performance of DS and typically developing children.

Vimercati et al. conducted research on 23 children with Down syndrome to perform a quantitative drawing evaluation [39]. Their results showed children with Down syndrome exhibit a psychomotor delay, which results in problems within motor sequences, with a low level of precision when drawing.

In 2014, Alesi et al. conducted a study with three children with Down syndrome that combined training with both professionals and at home. With the participation of parents, they stimulated the children, observing improvements in motor skills, reaction times, etc. [40]. ElMaksoud et al. conducted a study to investigate the effects of sensory motor sensory training [41]. Their results revealed that training is effective both individually and in groups to improve motor skills and quality of life.

Parhoon et al. [42] presented a study to investigate the influence of sensory stimuli and motor exercises on manual skills in children with mental retardation; specifically, in 24 children with Down syndrome, from five to seven years old, using sensory stimulation therapy. The results of the study showed positive effects on balance, coordination, gross motor skills, etc. Therefore, sensory stimulation in children with Down syndrome can improve their rehabilitation and gross motor skills.

Finally, the systematic review of the literature by Camargo et al. provided an exhaustive search for studies on the evaluation of manual dexterity of children and adolescents with Down syndrome [43]. They found a low number of publications on this subject, and not all of them presented the appropriate tools for evaluating motor skills. Thus, they concluded that more evaluation and intervention studies involving children and adolescents with Down syndrome should be carried out, because they exhibit slower manual dexterity.

In 2012, Berg et al. used Nintendo Wii games with a child with Down syndrome at home [44]. The objective was to improve manual dexterity by using these games to determine the effects of motor intervention. However, in the scientific literature, no studies have been found regarding the validation of CVMA in the population with DS involving stimulation using didactic exercises in environments of human-computer gestural interaction. This is the reason that motivates this work.

## 3. Materials and Methods

### 3.1. Gestural Platform: TANGO:H 

Gestural interaction platforms are one of the technologies that give space to the adaptation of computer interaction structures to people with a disability or pathology, such as DS. Visual-motor tracking has been studied from a technological perspective in the design of interactive systems. In order to make the learning process more efficient, the adaptation and attention load of the hand-eye coordination perspective that affects these systems have been taken into account [45]. Another paper [46] contains a compendium of research results on eye movement and its relationship with different disciplines, showing a special interest in the use of eye-tracking to stimulate higher-order cognitive functions.

In this area, this study made use of an open platform for gestural interaction called TANGO:H [47], which can be used to design teaching exercises (pairing, classification, and ordination) and physical rehabilitation and cognitive training activities using the KINECT sensor. KINECT is a three-dimensional (3D) camera developed by Microsoft. Unlike many predecessors, it is not based on a time-of-flight sensor, but on an infrared light pattern projected into the scenes. This yields great precision at a low cost. It is preferably used only indoors, or at least not under direct sunlight, which includes infrared energy as well. The KINECT sensor, however, does not come isolated from a midware layer of software. This layer includes a series of libraries that easily allow making real-time image segmentation, based on the camera image augmented with the depth information. In addition, other functions include the ability to recognize and track a human body, inferring the three-dimensional position of each of its most representative points, linked together to form a virtual skeleton. In our setup, the software benefits of tracking the user’s body position at any time, allowing for a rich interaction in different kinds of exercises, will be described below.

TANGO:H also keeps a record of interactions, gamification strategies, an objective evaluation of basic emotions, and personalization of interaction exercises. The platform has been designed to provide the user with a personalized learning experience, with transverse gamification, which has evolved with the incorporation of an intelligent system that, based on the historical data of the user’s interaction, recommends exercise levels (games), types of resources, and activities; the level of the challenge is increased, motivating the user to reach new levels of play based on their capabilities and interaction time. When designing the resources, it is suggested that they be of minimum complexity, and that this increases as progress is made. Therefore, the interaction resources are prepared by the expert (tutor) for the fulfillment of objectives in levels, varying in complexity, as required by the user when exceeding the limit (maximum value in time and precision) of the level. This perspective also helps guide activities during patient rehabilitation. That is, the game will start with simple activities for the patient, but with effort, and as the patient improves, the activities will become more difficult, which will require extra effort in more areas of his body. The awards ceremony is another of the gamification resources available to motivate the user to participate.

The platform also has an exercise editor that allows the user to customize the exercises and interaction variables: level, time, and range of action. Each exercise has its own structure, organized into steps, phases, and objectives; the latter being achievable in time of interaction through the various joints: head, shoulders, arms, hands, legs, and/or feet.

The principle on which the creation of this platform is based boils down to ensuring that the activities carried out through it are pleasant and not repetitive; that is, on providing the user with an experience that, from the first moment, creates in them the need to move forward and continue experimenting with the platform. For this reason, the platform incorporates a recommender system that provides the user with a new exercise, game, or activity, based on their user profile and interaction record with the platform. In addition to this, activities aimed at different types of players are provided. Furthermore, the implementation of a game level control system allows for improved adaptation and personalization of the exercises. For example, activities proposed to new players are basic, allowing them to adapt to the system while they learn. Activities proposed to more experienced players are more difficult, keeping them from becoming repetitive and posing new challenges to the user. This perspective also helps guide the activities during patient rehabilitation. That is, the game will start with simple activities for the patient, but with effort, and as the patient improves, the activities will become more difficult, which will require extra effort in more areas of his body. Moreover, TANGO:H includes several game modes, depending on the number of players or the type of gamified exercises. A game mode refers to the number of players (single or multiplayer) and the type of game, which can be sequential or simultaneous, competitive or collaborative. The modes can be classified based on the number of players that perform the exercise: single player and multiplayer. In single-player mode, one player will do the activity. In contrast, in multiplayer mode, the game is played by several users and the platform will provide several game modes to improve the experience.

The description of the TANGO:H platform and the different systems implemented to make the platform smart can be found below.

**Figure 1:**
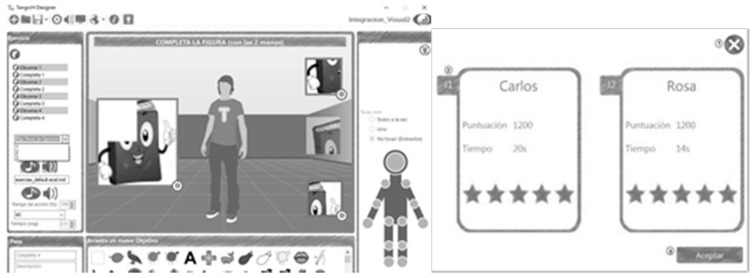
TANGO:H Designer and Game.

The TANGO:H platform is divided into two parts (Figure 1):

- TANGO:H Game, which focuses on the users and on interacting with them. Its purpose is to execute previously defined exercises/games that are selected by the users, as well as the game mode in which it will be carried out. The game uses the KINECT sensor to capture user gestures as a form of interaction.
- TANGO:H Designer, which focuses on the doctors or specialists, allowing them to design the exercises and lay out the steps and phases that the user must solve in order to satisfactorily complete the exercise. In addition, the designer also allows specifying the objectives of the exercise, so that the game is evaluated based on the completed objectives.

The exercises presented by TANGO:H consist of a set of objectives, with which the user has to interact gesturally. Thus, for TANGO:H, an activity consists of a set of exercises that can be physical, cognitive, or hybrid [48]. Exercises of the “physical” type rely on stimulating the user’s joints through the use of figures, called objectives. These figures are laid out on the screen by the designer, with colors showing the objectives and body parts that the user has to use at any given moment to satisfactorily complete the exercise. This way, the designer is able to set up a type of exercise aimed at users with reduced mobility or physical problems. The “cognitive” exercises base their functioning on the user’s cognitive development. These exercises aim to improve the perception, memory, learning, and reasoning abilities of users by using different pairing, ordering, and classification tasks. Finally, the “hybrid” exercises are configured in such a way that they do not follow any kind of pattern.

The activities are planned and implemented according to the objectives proposed by the expert. In all three types of activities, prior planning (academic or physical) is necessary. The team must then design all the resources (images, audio, relationships, sequences) and implement them in TANGO:H Designer. Interaction variables are configured such as: time, distractors, pairing or sequence relationships, audios, and prizes. In the end, the exercise is saved in a compressed file with a *.tica* extension, which will run in TANGO:H Game. It is in this platform that user control is implemented and activities are assigned to users. The three types of activities are developed and similarly implemented; the difference is in the objective set by the expert.

### 3.2. TANGO:H as a Cognitive Development Tool

**Figure 2:**
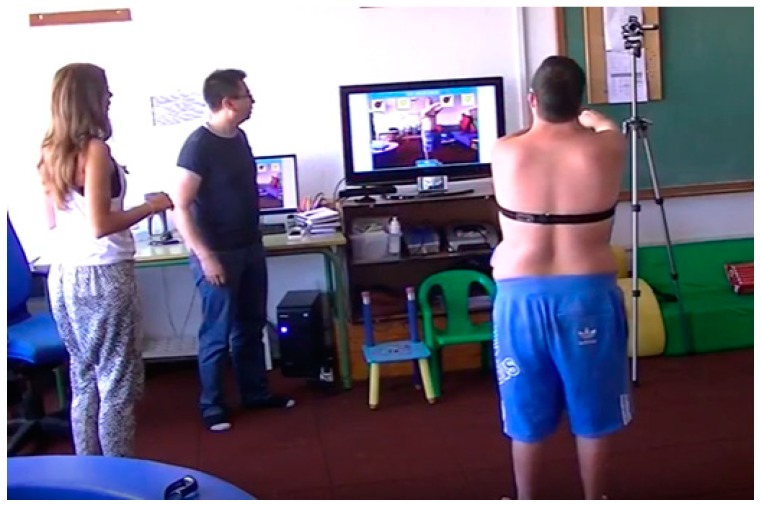
Uses of TANGO:H with individuals with Down syndrome in the Association Down Tenerife.

In this section, we detail how TANGO:H can be used as a rehabilitation tool, and in particular as a cognitive development tool. A rehabilitation plan might distinguish between different types of objectives. First, we might focus on recovering physical mobility and strengthening a muscle associated with some specific body joint. Alternatively, after neuronal/cognitive damage, the rehabilitation might include exercises to regain movement coordination, a cognitive process (Figure 2). In both cases, the patient should understand the concepts related to the process in question, so as to have a better understanding of the recovery process and the expected physical progress. The patient should also know the limitations facing them and how to choose the best strategies for cognitive development.

In each of the scenarios mentioned above, TANGO:H might have different interaction techniques, system limitations, and functionalities:

#### 3.2.1. Regarding Physical Movement

- The person should be facing the screen. Therefore, particularly relevant in this position are those movements that are done with the shoulder, elbow, and hips.
- Using TANGO:H, we can force the user to make either a pure joint movement or a functional movement. The former is a movement that involves moving a joint along only one rotation axis. Usually, this kind of movement affects only one muscle group. The functional movements seek to fulfill an action or reach a goal, and therefore, usually require a combination of movements with different joints, or a movement of the same joint along different axes.
- The difficulty of the movement is clearly related to the time available to complete it. The application can track how long the user is given to complete the exercises and movements, and therefore, the exercise parametrization.
- Furthermore, the precision that is required in the body’s position in order to recognize every movement as correct can be adjusted for every initial or end point of the movement. This can obviously change how hard or easy each exercise is. TANGO:H controls this setup by measuring the distance from the body to the projected goal on screen.
- Different tangible goals to reach are set up to appear sequentially on the screen. The way those sequences are built also directly defines the difficulty required to complete the exercise. Much more effort has to be made to reach goals located far from one another with respect to another situation in which one is close to the next.

#### 3.2.2. Regarding Cognitive Development

- Being able to see one’s own body represented on a screen, and the ability to draw on top of it the exact position reached by each joint, enhances body awareness and positioning, which is the first step towards better movement coordination.
- The tool also allows setting different goals to reach, sometimes simultaneously. In these situations, the movements to be done have to be coordinated; if they are not, the exercise is registered as incomplete.
- Therefore, this exercise involves pure coordination movement. In this case, not only time and accuracy, but also the number and distance of goals to be reached simultaneously, become a parameter for setting the exercise difficulty.
- The platform allows setting up ‘goals to avoid’. In this scenario, there are regions of the screen in which the user should never position specific parts of the body.
- This allows the exercise to force the user to find paths in space along which to move. This serves to improve movement coordination.

#### 3.2.3. Regarding New Knowledge Acquisition

- It is well-known that after being exposed to new knowledge for the first time, a person needs some time to internalize the information, after which it is useful to refresh this information periodically so as not to forget it. Those exact moments could be computed in advance and incorporated into the action plans for optimum improvement of the user’s learning curve.
- TANGO:H allows introducing information capsules inside the exercises. That information may or may not be related to the cognitive development itself. At certain points, it could be useful to schedule refresher exercises as mentioned above, and in other cases, the best strategy might be to distract the user’s attention from the rehabilitation process. This could result in increasing the activity periods during which the user is willing to use the application, reducing mental fatigue and potentially the stress the person experiences. At the same time, after the distraction activity, the attention and intensity of the ensuing activities would increase significantly.

#### 3.2.4. Motivation

- The application can display screenshots to exhibit how the user interacted with the system on previous occasions. This allows visualizing the progress that might have taken place in a certain period of time, and incentivizes the user to continue using the platform.
- There is also the ability to personalize ‘themes’. This ‘theme’ can be selected based on a user’s interests and personal preferences. The exercises use images to help the user locate the goals to be reached with each movement, sounds are played, descriptions are displayed, etc. All these elements can be used to build a narrative around certain topics, or simply to contextualize the exercise with items that are appealing to the user. This contextualization might also depend on the user, task, and difficulty level, and might provide additional motivation to the user to be or stay involved with the tasks.

#### 3.2.5. Recommender System

The recommender system incorporated in TANGO:H can be adapted naturally to be used in the context of CVMA development. The only difference in the way the system works with respect to the recommender system described earlier is the terminology. First, the skills to be developed are not skills related to an academic curriculum, but to the CVMA process. In the case of physical rehabilitation, each skill will be defined as a joint and a mobility range to be exercised. The type of movement to be performed within the mobility range, for example, muscle extension, will define different types of activities for that skill. In the case of cognitive development, skills are defined in terms of the combination of movements and joints that are involved.

Furthermore, the recommender system should define a set of difficulty levels so that the activities can be adapted to each user’s skills. These difficulty levels can be defined using the exercise parameters that were provided when defining TANGO:H as a CVMA tool. They include:

- Time available to complete each movement.
- Movement accuracy for goal recognition.
- Goal resizing.
- Objects to avoid on-screen between the desired goals.
- Activity duration.
- The number of movements involved in coordination tasks.

## 4. Validation

In order to evaluate the efficacy of our gestural platform, we have evaluated how this kind of interaction impacts the cognitive visual-motor abilities of individuals with Down syndrome. The design of this experiment follows the single-case experimental design (SCED) approach. This term refers to experimental methods used to test the effect and efficacy of an intervention with a small number of patients (usually one to three) when populations are small or heterogeneous, in a clinical setting, etc. According to this approach [49], a case can be a person or a group of people with a single characteristic on which the study will be focused. SCED relies on observation with visual analysis, repeated measurements, internal and external validity, etc. [50,51,52]. SCED can also be used to evaluate rehabilitation efficacy [53]. The study involved six participants with SD, divided into two small groups of three. It is common to experience difficulties in recruiting users with disabilities for studies. According to Lazar et al. [54], for research focusing on users with disabilities, it is generally acceptable to have 5–10 users with a specific disability take part in a study.

In this study, the hypotheses to validate are the following:

- hA0: Visual-motor memory ability in students with Down syndrome improves after stimulation with gestural interaction using the KINECT sensor.
- hB0: The change in the visual-motor memory ability in students with Down syndrome after being stimulated with gestural interaction is greater than in a group with typical classroom stimuli.

The evaluation was arranged into three phases (pre-test, test, and post-test) with different methods and instruments (Table 1). Questionnaires, interviews, and video records were employed, and we also analyzed the TANGO:H logs and videos.

In the pre-test, the structured observation can be used to evaluate the skill level of the participants. We also conducted a semi-structured interview with the experts to determine the cognitive and motor skill level, available in the academic record, and in the results of the ITPA test applied beforehand. With the results of both evaluations, we obtained the participants’ profile (Table 2). Afterwards, we designed the cognitive exercises taking into account the participants’ profile.

In the test phase, we carried out a structured observation during the session using a form. The sessions were recorded on video as well. Different variables (type of resource, successes, distractors, time required to meet the objectives, emotional state during the entire interaction) on the interaction with the system were captured, as well as the achievement of the goals and skill level.

In the post-test phase, the skill level was evaluated based on the opinion of the experts as to the changes observed in the student participants, and also based on the percentage of progress in the acquisition of the abilities and skills proposed. In addition, the videos of the sessions were analyzed and evaluated. A codification was done prior to the data analysis and evaluation.

In the following sections we will focus on two main instruments (IPTA and TANGO:H) and the observational method. The variables studied will be discussed in the Results section.

### 4.1. Participants

The study was conducted with the approval of the Ethical Board of the Tenerife Down Association. The sample was selected from the population of the Down Tenerife Association, which has 56 DS students. Written informed consent was obtained from the participants’ parents prior to enrollment. The sample (*n* = 6, two girls and four boys) was divided into an experimental group (EG, *n* = 3, one girl and two boys) and a control group (CG, *n* = 3, one girl and two boys), with the former being stimulated by using the TANGO:H with gestural interaction, while the control group worked on a daily basis in the classroom without using gestural stimulation.

Regarding the age, it is important to note that children with Down Syndrome have two age classifications: chronological age and mental or cognitive age. A Piaget study identified four development stages for acquiring cognitive skills: sensorimotor, preoperational, concrete operations, and formal operations [55]. However, different works suggest that the process takes much longer for Down Syndrome children to complete [56,57], and that children with Down syndrome may function at a mental stage below their actual chronological age [58]. The cognitive level is the relevant guide to their performance, not chronological age. As a result, in these cases, we have to focus on the mental age of a child with Down syndrome, rather than on their chronological age [59].

The inclusion criteria for this study were the following: children diagnosed with DS, high interpersonal intelligence level and high communication abilities, and cognitive age greater than five years old. Regarding the exclusion criteria, we considered children without DS, low interpersonal intelligence, low communication abilities, and cognitive age below five years old.

### 4.2. Methods and Instruments

As stated earlier, in this study, we applied two main instruments: the Illinois Test for Psycholinguistic Abilities (ITPA) [60] and various stimulation exercises using TANGO:H. Furthermore, as qualitative methods we used observations and interviews, recorded and analyzed the logs of the platform as well as the videos of all the sessions. Below is a description of both the instruments and methods used in this study.

#### 4.2.1. Illinois Test for Psycholinguistic Aptitudes (ITPA)

The Illinois Test for Psycholinguistic Aptitudes (ITPA) was used to evaluate the psycholinguistic functions involved in the communication process and establish variables to determine the degree of disorder in various areas of learning. It is composed of 12 sub-tests organized into categories [60]. As we mentioned in the Introduction section, four sub-tests were selected: visual comprehension, visual association, visual integration, and visual-motor sequential memory.

ITPA has provided the methodological basis for numerous scientific studies, several of them involving individuals with DS [60,61] and pathologies such as Smith-Magenis syndrome [62]. One study examined the performance of sequential bilingual children with and without Specific Language Impairment [63].

As in our study, several researchers have applied some of the ITPA subtests, selected and adapted them to the problem with relevant results such as those obtained by Huber et al. [64], where 3–10% of all children with specific language processing deficits can be identified by the Auditory Integration sub-test; this sub-test has also been used to relate the linguistic characteristics of twins in a Japanese adaptation of the ITPA [65].

Its application has been used to contrast the normal development of language in groups [66] and to evaluate their linguistic abilities after stimuli with music [67]. Seung et al. [68] used ITPA to examine whether hearing status affects the performance of short-term verbal-auditory memory in DS; in addition, the psycholinguistic age obtained with ITPA has been one of the parameters for selecting the sample—a population with intellectual disability [69]. Similar to Seung et al. [68], our goal is to extend the use of ITPA as well, which is used in the Down Tenerife Association to establish the psycholinguistic age of all the students.

Within the ITPA sub-test group [70], references specifically to visual-motor memory have been selected, which mainly require the ability of visual discrimination. Visual comprehension corresponds to the ability to obtain information from symbols and visual patterns; visual association corresponds to the ability to analyze conceptual relationships that are presented visually, such as discovering sequential patterns between objects; visual integration corresponds to the ability to identify known symbols from an incomplete representation of them; and visual-motor sequential memory corresponds to the ability to reproduce, after a stimulus, sequences of symbols (words, numbers, letters) presented visually. These areas are required to implement and develop cognition, associative memory, sensory integration, and visual discrimination skills, through exercises such as visual analogies, ordering simple symbols, assembling puzzles, developing graphic algorithms by organizing sequences of activities, association by color, etc. These activities have been adapted to brief exercises that can be used to systematically stimulate the skills described above.

In our case, four ITPA subtests were applied that correspond to the validation of visual-motor memory: visual comprehension, visual association, visual integration, and visual-motor sequential memory. The test was applied individually by a speech therapy professional from the Down Tenerife Association, following the protocols established in the ITPA manuals for application and correction.

#### 4.2.2. TANGO:H Gestural Platform: Exercises

**Figure 3:**
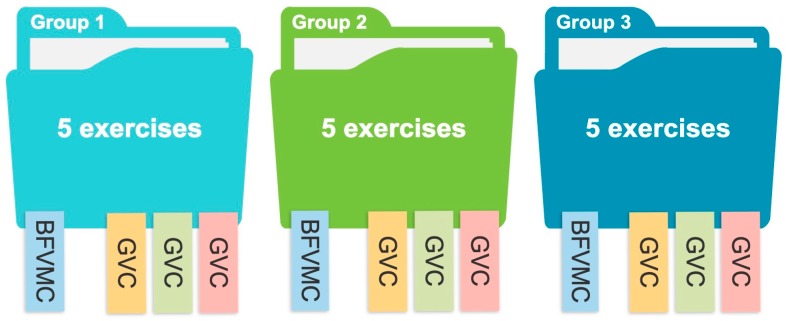
Each group of exercises stimulates one of the Basic Factors of Visual-Motor Cognitive (BFVMC) aptitudes, and the three Groups of Visual Comprehension (GVC).

Three groups of exercises were designed in TANGO:H Designer with five exercises each (see Figure 3), where a pattern to be followed is presented initially and on the next screen a response related to the previous pattern is requested.

Each group of exercises stimulates one of the basic factors of visual-motor cognitive aptitudes (Table 3), and the three groups of visual comprehension. The exercises were applied iteratively over a month (four lessons), with a weekly lesson lasting approximately 20 min. Each lesson can contain as many exercises as the professional decides.

### 4.3. Observation

To evaluate the progress between each lesson longitudinally, a semi-structured observation was made in all the working lessons with the EG, for each individual and session. The observation was made by the expert speech therapy teacher in (E1), psycho-rehabilitation (E2), language class teacher (E3), and a computer science researcher (E4). The participants were observed, directly and individually, with an average group assessment and by consensus. The group of experts who made the observation worked together throughout the period of preparation of the experiment, the experimentation, and analysis of results. The knowledge that each one of the experts has about the individuals constitutes a significant contribution to the study, since they can relate each fact from events and attitudes of the individual. The following variables were measured:

- From the TANGO:H platform: the time to complete the task (per session and lesson) and the interaction errors (per session and lesson).
- Observation: Fluency (from Likert scale [1: very low–5: very high]); understanding of the pattern shown (from Likert scale [1: very low–5: very high]); and confidence-security level in responses (from Likert scale [1: very low–5: very high]).

### 4.4. Results

**Figure 4:**
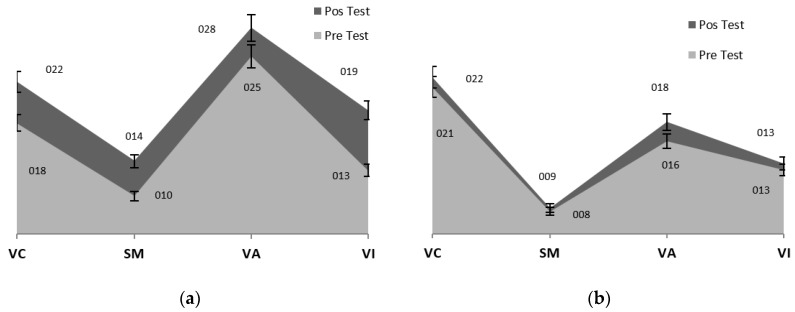
Pre/Post-Test Results from the ITPA sub-test in EG and CG. (**a**) Pre/Post Test EG (Error bar 5%); (**b**) Pre/Post Test CG (Error bar 5%). (VC = Visual Comprehension; SM = Sequential Memory; VA = Visual Association; VI = Visual Integration.)

Table 4 shows a summary of the results of the four ITPA sub-tests applied. The variation in the Direct Score is considerable in all the subtest of the EG (Figure 4a) and reduced in CG (Figure 4b). Only in two cases were there changes in EG, in Visual-Motor Sequential memory (VMS) of EG_1 and in Visual Comprehension (VC) of EG_2; likewise, despite not having been part of the stimulation program, in CG_3 improvements were seen in the results of all the sub-tests, and in CG_2 in Visual Association (VA).

The graphical reports present the variation between the means of the direct score of the four sub-tests, showing the upward trend between pre-test and post-test, which is greater in EG (Figure 3a). This improvement in the direct score after stimulation with teaching exercises is visible in all the EG sub tests, being higher in Visual Integration (*x*_2_ − *x*_1_ = 6.33) and lower in Visual Association (*x*_2_ − *x*_1_ = 3.00).

**Figure 5:**
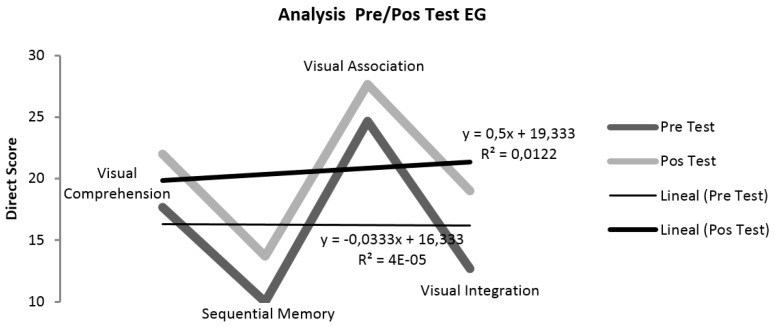
Pre/Post-Test Analysis in Experimental Group (EG).

To obtain the results that confirmed hA0 in the EG, the Student T-statistic was applied for related samples, as was a descriptive analysis based on measures of central tendency (mean and standard deviation). As Table 5 shows, in none of the cases is the difference statistically significant, but this may be due the low number of participants (*n* = 3). However, as Figure 5 shows, there are improvements from pre- to post-test in Visual Comprehension, Visual Association, Visual Integration, and Sequential Memory in the EG.

In the case of hB0, the Student T-statistic is applied for independent samples between the differences of the means of the pre-test and post-test of both groups, and as in the previous case, the variation in Paired Differences (PD) of the four sub-tests is compared (see Table 6). In all cases, we see that despite a visible difference, it is not statistically significant, with the p-value of all the pairs exceeding the expected 95% significance; Levene’s Test is required in this type of statistical comparison, as it measures the equality of variances as a precondition for the application of t-student in independent samples.

**Figure 6:**
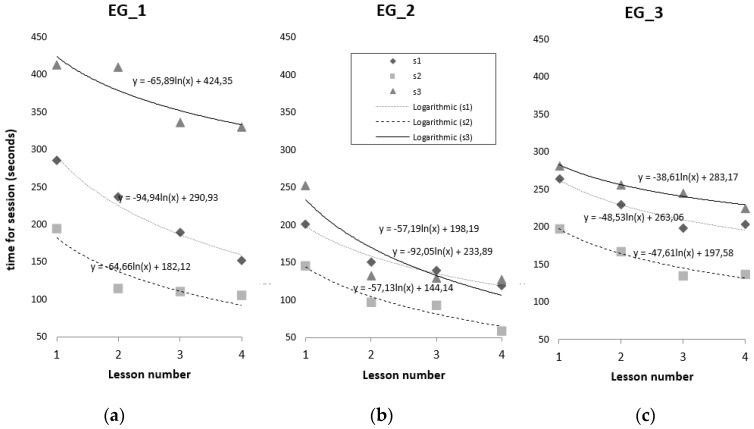
Variation in the time that each EG individual needs to complete tasks. (**a**) EG_1; (**b**) EG_2; (**c**) EG_2.

In every case, the time that each individual needed to complete the stimulation task (Figure 6) tends to decrease between each lesson (variation = 1 week). To obtain the equation for the behavior of the variable, a logarithm function is fitted to the data. In all three cases and for all three types of exercises, there is a decreasing variation, which indicates that the individual acquires skill and knowledge about the exercise. This variation tends to be greater between the first and second lessons.

The behavior of the ‘errors made during the stimulation’ variable has a curve whose behavior is similar to that of the ‘time’ variable; that is, its value decreases with every stimulation lesson. The variation in the errors made between the first and second lessons is larger than it was for the time variable, with the exception of session 1 in EG_1, which remains constant during the four lessons (*y* = 2). The average difference between the first and last lessons for each individual is: EG_1 = 101 s; EG_2 = 98 s; EG_3 = 59 s. In every case, this difference is higher in the exercises proposed in session1, on visual association.

The observation of the experts (Table 7), despite being considered a subjective assessment, draws on their knowledge to enhance our understanding of the impact of the stimulus on the skills of the individual. All the variables exhibit growth, if the first and last lessons are compared. The individual with the best performance is EG_2, who enjoyed the whole interaction and mastered the platform and learning resources, reaching five on the Likert scale in the three variables considered. EG_1 shows a change with a tendency to improve (from 2 to 4). In the case of EG_3, an improvement is visible; however, the learning curve is not constant. In this case, we must note that the emotional changes influenced the final lesson, which caused a decrease between the third and fourth lesson.

## 5. Discussion

The CVMA has been validated by several researchers as a sensible point for achieving better learning in people with DS. The main goal of this study was to evaluate if the gestural interaction with the KINECT sensor improves the cognitive visual-motor abilities in people with Down syndrome. In this sense, the results are satisfactory, given the considerable improvements observed in the EG (+3.00 to +6.33 Direct Score). However, the t-Student statistical results for related samples in hA0 and independent ones in hB0, with a confidence interval of 95%, did not show a considerable variation, thus, the hypotheses of equality of means are accepted for both cases. Regarding hA0, visual-motor memory capacity in students with DS did not improve after stimulation using didactic strategies in an environment of computer gestural interaction; regarding hB0, the variation in the visual-motor memory capacity in students with DS after stimulation using cognitive exercises in an environment of gestural computer interaction is greater than that of a group with typical classroom stimuli. There is a growing variation in the measurements made in some parameters for the CG, which corresponds to the daily development of each child due to the stimuli received from their environment. It should be noted that the students participating in this research attend general schools, and in the Tenerife Down Association, they receive complementary stimulation. Note that both groups (CG and EG) received this complementary stimulation, but the CG did not receive stimulation with TANGO:H using the KINECT sensor, which was an additional stimulation to the EG.

The limitations of the study are, first, that it was limited to an objective assessment from the subtests of the ITPA test involving visual-motor cognitive abilities before and after the stimulation through the TANGO:H gestural interaction platform and the exercises specifically prepared for this study. Moreover, the study sample was limited to six individuals (CG = 3, EG = 3), whose results are assessed in the experimental single-case study methodology. It is not the objective of this study to extrapolate these results to the general population with DS, but to contribute with a scientific evaluation of a technological gestural stimulation platform to improve the quality of life of individuals with DS.

The sub-tests of ITPA have allowed for a methodological corroboration of the variations that students have had regarding CVMA. The ITPA [60], as well as TONI [36], make use of figures and shapes to validate the student’s degree of cognitive and visual-motor development [71,72]. It is possible to affirm that the ITPA sub-tests are adequate for validating the change in CVMA after stimulation using didactic exercises in environments of human-computer gestural interaction.

The TANGO:H interaction platform allowed us to design adequate exercises for the EG. This research corroborates the work done to validate the TANGO:H platform [7,61], expanding its applicability to the stimulation of CVMA in children with DS. In addition, given the stimulation times per session, the quantity and design of the exercises were adequate.

## 6. Conclusions

In this paper, we have described the TANGO:H platform, where the user interacts with the system by using their body. The system uses a KINECT sensor to recognize gestures, requiring no physical contact with traditional control systems, and takes into account the results obtained in the study carried out on the effectiveness as a cognitive visual-motor rehabilitation tool. The cognitive visual-motor skills improved in all the sub-tests applied to the EG, which suggests applying the exercises to a larger population, as well as increasing the stimulation phase. Working with the ITPA sub-tests allowed us to sustain the work in a standardized methodology, and to evaluate the effectiveness of the gestural stimulation with the KINECT sensor.

The TANGO:H platform allowed us to design and implement digital exercises for gestural interaction, adding to its toolkit the stimulation of cognitive visual-motor skills in individuals with DS. The gestural platform allows stimulating visual-spatial memory in individuals with DS, yielding superb results in every case.

Interaction times in learning spaces based on gestural interaction devices decrease significantly between lessons on the same thematic contents. This involves improving reading skills in individuals with DS. These results represent a real and significant contribution to the related sciences: the psychology of learning, pedagogy and human-computer interaction.

Users with DS quickly learn to interact with the didactic resources of the gestural interaction platform. This is evidenced by the continuous decrease in the non-cognitive errors exhibited in the initial sessions; they also tend to disappear as the experimentation process progresses, with improvements in their gestural interaction skills.

The graphic reports show that the CVMA improved in all the subtests applied to the EG. Thus, we suggest applying the resources to a larger population, as well as increasing the stimulation phase. In addition, the results of the observational study carried out by the professionals of the institution exhibit a considerable change between one study group and another.

In the future, a study of those parts of the body that are most difficult for the user to move could easily be performed and presented afterwards. Consequently, the specialist would be able to observe those areas in which the patient is improving or where the treatment could have a greater impact. In addition, since the KINECT sensor provides the ability to track the user’s face, the use of gesture capture can be used to study the user’s satisfaction with the application or be used to create exercises for those with other problems, such as facial paralysis. Thus, they could do exercises that could help them regain control of their facial movements.

From the technological perspective, it would be of great interest to use other physiological and sensory variables in order to determine the change that the individual undergoes during the stimulation processes with the gestural interaction with TANGO:H.

It is also feasible to extend the study to individuals without DS to conduct a comparative study between these two populations. These results would be comparable to those obtained in the study.

## Figures and Tables

**Table 1:** Different phases, methods, and instruments used in the study.

Phase	Method/Instrument	Actors
**Pre-test**	Semi-structured interview TANGO:H (Designer) ITPA Test	Experts Teachers Individuals with DS
**Test**	Structured observation (Session) Platform TANGO:H	Researchers Teachers Individuals with DS
**Post-Test**	Semi-structured interview TANGO:H (Designer) ITPA Test	Teachers Individuals with DS

**Table 2:** Participants in the study.

Groups		Demography	
		Chronological Age	Cognitive Age
**Experimental (EG)**	Median	25	7
	SD	4.97	0.82
	Min	18	6
	Max	29	8
**Control (CG)**	Median	13.67	6.67
	SD	4.11	1.69
	Min	9	5
	Max	19	9

**Table 3:** Exercises for cognitive visual-motor stimulation.

Exercises	Description	Type
**(a) Sequence of exercises for visual association** 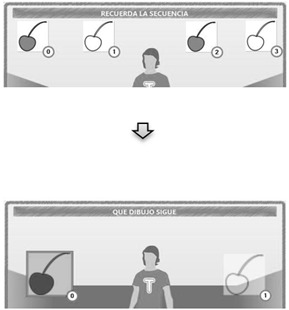	Expressing the pattern of a sequence, selecting each element correctly after inferring the existing pattern; the student goes through all the elements of the pattern, placing both hands on each object for two seconds. In the first step the sequence pattern is shown (top), and in the next step the student must discover the sequence from similar objects (bottom).	Visual Association
**(b) Exercise for visual integration** 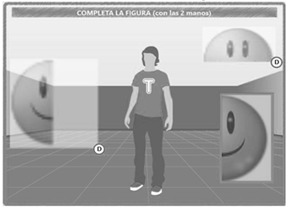	First, an image is shown that the student must select with by using both hands for two seconds, trying to remember it; the previous image is presented in the next step with a space to complete as a puzzle, whose missing piece must be chosen from the options on the right.	Visual integration
**(c) Exercise of Sequential Visual-Motor Memory** 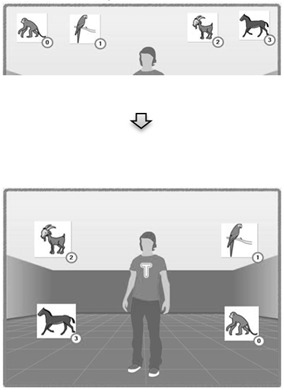	The objects are arranged in a certain order at the top. They have to be selected by placing both hands on each object for two seconds; then, the figures are shown out of order, and the student selects them one by one following the previous order. The sequential steps were done first with numbers, then with images of animals that are known to students. The complexity increases as it progresses.	Sequential Visual-Motor Memory

**Table 4:** Summary of results ITPA in EG and CG.

VC = Visual Comprehension; VA = Visual Association; VMS = Visual-Motor Sequential Memory; VI = Visual Integration; EPL = Psycholinguistic Age											
	TANGO:H Stimulus					Pre-test			Post-test		
Students	Lessons	Exercise 1	Exercise 2	Exercise 3	Total Time (mm:ss)	Direct Score		EPL	Direct Score		EPL
EG 1	4	4	4	4	76:42	VC:	11	4-0	VC:	19	5-6
						VMS:	12	7-2	VMS:	12	7-2
						VA:	26	7-4	VA:	28	7-10
						VI:	12	3-0	VI:	14	3-1
EG 2	4	4	4	4	71:36	VC:	25	7-10	VC:	25	7-10
						VMS:	10	6-3	VMS:	19	10
						VA:	30	8-7	VA:	31	8-11
						VI:	12	3-0	VI:	22	4-5
EG 3	4	4	3	4	73:21	VC:	17	5-0	VC:	22	6-7
						VMS:	8	5-9	VMS:	10	6-3
						VA:	18	5-8	VA:	24	6-11
						VI:	14	3-1	VI:	21	3-9
CG 1	0	0	0	0	0:0	VC:	21	6-2	VC:	21	6-2
						VMS:	10	6-3	VMS:	10	6-3
						VA:	15	4-9	VA:	15	4-9
						VI:	12	3-0	VI:	12	3-0
CG 2	0	0	0	0	0:0	VC:	25	7-10	VC:	25	7-10
						VMS:	10	6-3	VMS:	10	6-3
						VA:	16	5-1	VA:	20	6-3
						VI:	13	3-1	VI:	13	3-1
CG 3	0	0	0	0	0:0	VC:	18	5-3	VC:	21	6-2
						VMS:	5	4-9	VMS:	6	5-1
						VA:	16	5-1	VA:	18	5-8
						VI:	13	3-1	VI:	15	3-2

**Table 5:** Central tendency statistics and relational T-Student in EG for ITPA.

Paired Samples Statistics						Paired Differences			t	Significance Level (Two-Tailed)
Sub test ITPA		Mean	N	Standard Deviation	Standard Error Mean	Mean	Standard Deviation	Standard Error Mean		
**Pair 1**	Visual Comprehension Pos	22.00	3	3.000	1.732	4.333	4.041	2.333	1.857	0.204
	Visual Comprehension Pre	17.67	3	7.024	4.055					
**Pair 2**	Sequential Memory Visual Motor Pos	13.67	3	4.726	2.728	3.667	4.726	2.728	1.344	0.311
	Sequential Memory Visual Motor Pre	10.00	3	2.000	1.115					
**Pair 3**	Visual Association Pos	27.67	3	3.512	2.028	3.000	2.646	1.528	1.964	0.188
	Visual Association Pre	24.67	3	6.110	3.528					
**Pair 4**	Visual Integration Pos	19.00	3	4.359	2.517	6.333	4.041	2.333	2.714	0.113

**Table 6:** Test applied to the control group.

		Levene’s Test				t-test for Equality of Means				
		F	Sig.	t	df	Sig. (two-tailed)	Mean Difference	Standard Error Difference	95% CI of the Difference	
									Lower	Upper
**VC**	Equal variances assumed	1.750	0.256	1.313	4	0.259	3.333	2.539	−3.715	10.382
	Equal variances not assumed			1.313	2.711	0.289	3.333	2.539	−5.256	11.923
**SMV**	Equal variances assumed	8.522	0.043	1.213	4	0.292	3.333	2.749	−4.298	10.965
	Equal variances not assumed			1.213	2.060	0.346	3.333	2.749	−8.171	14.838
**VA**	Equal variances assumed	0.571	0.492	0.522	4	0.629	1.000	1.915	−4.316	6.316
	Equal variances not assumed			0.522	3.723	0.631	1.000	1.915	−4.476	6.476
**VI**	Equal variances assumed	3.028	0.157	2.335	4	0.080	5.667	2.427	−1.071	12.404
	Equal variances not assumed			2.335	2.324	0.127	5.667	2.427	−3.495	14.829

VC = Visual Comprehension; VA = Visual Association; SMV = Sequential Visual Memory; VI = Visual Integration. Sig. = Significance level (typically 0.05). df = degrees of freedom. CI = Confidence Interval.

**Table 7:** Variables observed by experts.

Participant	Variable	Lessons (Likert Values: 1 low–5 high)			
		L1	L2	L3	L4
EG_1	Fluency	2	3	4	4
	Pattern comprehension	2	3	4	4
	Confidence level	2	4	3	3
EG_2	Fluency	4	5	5	5
	Pattern comprehension	3	4	5	5
	Confidence level	4	5	5	5
EG_3	Fluency	2	3	4	3
	Pattern comprehension	1	2	3	3
	Confidence level	1	3	3	2
